# The Use of Multimodal Perineural Adjuvants in Pediatric Peripheral Nerve Blocks: Technique and Experiences

**DOI:** 10.7759/cureus.23186

**Published:** 2022-03-15

**Authors:** Sampaguita P Tafoya, Sundeep S Tumber

**Affiliations:** 1 Pediatric Anesthesia, Shriners Hospitals for Children, Northern California, Sacramento, USA

**Keywords:** nerve block, regional anesthesia, child, perineural, adjuncts, adjuvants

## Abstract

Background

Postoperative pain management in pediatric population can be very challenging. How to prolong the duration of single-injection peripheral nerve blocks has been widely discussed. Multiple medications are under investigation to accomplish this, yet data specifically focused on their use in pediatric peripheral nerve blocks are limited.

Methods

Anesthetic electronic medical records were queried for any instances where adjuvant drug(s) were used in peripheral nerve blocks during a two-year period at a pediatric surgical specialty hospital. These included buprenorphine, clonidine, dexamethasone, and dexmedetomidine.

Results

Out of 1,845 blocks placed during the study period, 1,148 (62.2%) utilized perineural adjuvants. Buprenorphine as a sole agent was the most common choice (49.5%), followed by buprenorphine and dexmedetomidine combined (39.9%), dexmedetomidine alone (10.1%), and the rare combination of all three drugs, buprenorphine, dexmedetomidine, and dexamethasone (0.5%). The mean dose of buprenorphine given was 3.6 mcg/kg total, 2.8 mcg/kg/block. The mean dose of dexmedetomidine given was 0.9 mcg/kg total, 0.6 mcg/kg/block. The mean dose of dexamethasone utilized was 2 mg total, 1 mg/block.

Conclusions

This report examined one institution’s use of multimodal perineural adjuvants in over 1,000 pediatric peripheral nerve blocks. Buprenorphine was the agent most commonly used to prolong the single-injection peripheral nerve block. This highlights the need for future prospective trials evaluating efficacy and safety.

## Introduction

Despite advancements in multimodal pain therapies, postoperative pain in pediatric patients remains a challenge in as high as 40% of cases [1]. Safe management of this pain is crucial and often relies on regional anesthesia techniques. Peripheral nerve blocks effectively block pain sensation, allowing a child to be exposed to less systemic anesthetic agents throughout surgery, to have more effective analgesia, and to experience a reduction in opioid medication exposure, which carries significant risk [1].

The ideal peripheral nerve block must provide long-acting analgesia to adequately treat postoperative pain. Local anesthetics traditionally used in nerve blocks offer 4-12 hours of pain relief [2] depending on the block location, drug concentration, and volume injected. This duration is often insufficient when considering that postoperative pain associated with moderate-major surgery typically lasts 24-72 hours [2]. Recent use of adjuvant medications with local anesthetics in peripheral nerve blocks has increased in hopes of prolonging block duration. When adjuvant medications are incorporated, analgesia equivalent to that of catheter-based techniques may be possible [3].

Regional anesthesia techniques must be safe, efficient to place, and palatable to patients, parents, and surgeons. Continuous catheters offer long-duration analgesia, yet pediatric surgery often does not warrant the use of complicated, costly, and resource-intensive techniques [4]. Catheter issues such as bacterial colonization [5], dislodgement, kinking, malfunction [6], and up to a 50% failure rate [7] cannot be ignored. Young children may pull on catheters, parents may be uncomfortable managing catheters at home, and the requirement for postoperative casts after orthopedic surgery may make the use of catheters untenable. Rather, a longer-duration single-injection nerve block may be the ideal solution. Low concentrations of local anesthetics, which can allow for postoperative neurologic assessment and the breakthrough of compartment syndrome-mediated pain, make the addition of adjuvant medications especially attractive.

Many medications, in multiple different drug classes, are being explored for adjuvant use with local anesthetics. The ideal additive medication(s) should be supported by meta-analysis data, have a known potential perineural mechanism of action, have a tolerable side effect profile, and be available in a preservative-free formulation [4]. Investigations in adults determining the optimal adjuvant medication(s) and their use in combination are conflicting or sparse. Rare studies have been performed in pediatric patients, yet there exists only reports of single-agent use [8] and use in spinals and caudals rather than specifically in peripheral nerve blocks. Specific dosage recommendations for multimodal peripheral application [2] have not been established.

The most robust evidence for adjuvant use specifically to pediatric regional anesthesia involves the α_2_-agonist clonidine and highly selective α_2_-agonist dexmedetomidine [8,9]. Initial investigations in children began with the use of neuraxial clonidine, followed by use in peripheral nerve blockade. Adult investigations have swayed toward dexmedetomidine’s greater efficacy as compared to clonidine [10] and shown its potential for approximately 5 additional hours of analgesia [11]. A clear difference in effect from perineural administration as compared to intravenous administration has been shown in adults [12], but is still in question in pediatrics.

Buprenorphine is a mixed opioid agonist-antagonist that displays partial mu agonist and weak kappa antagonist activity. It is the least studied adjuvant agent in the pediatric literature, but has been shown to have the most robust peripheral nerve block analgesia prolongation of 8 hours in the adult literature [13]. It has the potential to cause increased postoperative nausea and vomiting (PONV) [13], yet the rate of this increase in the setting of double or triple antiemetic prophylaxis has not been established.

Dexamethasone is a corticosteroid commonly used during general anesthesia for PONV prophylaxis and in pediatric anesthesia particularly for the reduction of intubation-associated airway edema. It has demonstrated peripheral nerve block prolongation when used as a perineural adjuvant in adult regional anesthesia [14]. It has also been shown to be of benefit when combined with buprenorphine and/or clonidine [15,16]. However, its perineural use in children has not been studied [17]. In both the adult and pediatric literature, its benefit over that of intravenous administration is yet to be clearly shown [17,18].

Based on an extrapolation of the adult anesthesia literature, along with the scant pediatric literature that exists on this topic, and the known safety of each of these agents in vivo [19], our group of pediatric anesthesiologists began using adjuvants in peripheral nerve blocks in 2018. These included buprenorphine, clonidine, dexamethasone, or dexmedetomidine injected with traditional local anesthetics. This report retrospectively accounts on our department’s technique and experience with 1,148 peripheral nerve blocks placed utilizing multimodal adjuvant agents.

## Materials and methods

Approval was granted for this investigation by the WGC IRB on July 6, 2021 (IRB Study No. 1312780). In 2018, our pediatric anesthesia group began perineural adjuvant use. Anesthetic records were reviewed from a two-year period (May 1, 2019 to May 31, 2021). A query of the anesthetic electronic medical record (Anesthesia Information Management System, Cerner) was performed. All instances where a dose of buprenorphine, clonidine, dexamethasone, or dexmedetomidine was administered with the route listed as “block” or “perineural” were included and drug combinations and doses were analyzed.

All additives were combined with 0.125-0.5% ropivacaine, dosed at the anesthesiologist’s discretion. All blocks contained 1:200,000 epinephrine, as a marker for intravascular injection. Concentrated, preservative-free additive formulations were used in all cases (buprenorphine 300 mcg/1 mL, clonidine 1000 mcg/10 mL, dexamethasone 10 mg/1 mL, dexmedetomidine 200 mcg/2 mL).

All nerve blocks were placed at Shriners Hospitals for Children, Northern California, a free-standing surgical children’s hospital whose primary focus is orthopedic and burn care. Blocks were placed by a group of eight board-certified pediatric anesthesiologists with a minimum of three years of intensive pediatric regional experience using ultrasound guidance. A dosing reference sheet (Table 1) was created after review of the adult and pediatric literature in 2018. Actual drug doses and combinations of agents were determined on an individual case basis by the anesthesiologist providing care, taking into consideration the number of blocks required, patient comorbidities, and individual pain management needs.

**Table 1:** Pediatric peripheral nerve block adjuvant dosing used by one institution. Drug doses and application are off-label. Max, maximum dose.

Shriners Northern California Department of Pediatric Anesthesia	
Peripheral Nerve Block Additives Dosing Reference Sheet	
1st– Buprenorphine	
1 Block: 2-4 mcg/kg, max 300 mcg	>1 Block: total max 4 mcg/kg or 450 mcg
+2nd – choose one of the following:	
Dexmedetomidine	Clonidine
0.5 mcg/kg per block	1 mcg/kg per block
Total max 1 mcg/kg or 100 mcg	Total max 2 mcg/kg or 150 mcg
+3rd – Dexamethasone	
Infant: 0.015 mg/kg	Max 1 mg per block
School-aged: 0.5 mg per block	Total max 4 mg
Adolescent: 1 mg per block	Consider reducing IV dose

## Results

Over the two-year study period, 1,845 single-injection peripheral nerve blocks were placed in 1,461 pediatric patient cases at our institution. The distribution of block types by patient age group is displayed in Table 2. In 1,148 (62.2%) of these blocks, an additive medication (buprenorphine, dexmedetomidine, or dexamethasone) was given. Clonidine was not administered in any peripheral nerve block during the study period.

**Table 2:** Distribution of pediatric peripheral nerve blocks by age and type. Lat Fem Cutaneous, lateral femoral cutaneous; PENG, pericapsular nerve group; TAP, transversus abdominal plane; IPAK, infiltration between the popliteal artery and capsule of the knee.

Type of Block	Age <1 year	Age 1-<3 years	Age 3-<10 years	Age 10-<18 years	Total
Adductor canal	1	8	110	453	572
Popliteal sciatic	3	35	150	355	543
Femoral	6	34	65	103	208
Infraclavicular	1	46	62	95	204
Fascia iliaca			27	48	75
Axillary	2	8	18	33	61
Erector spinae			11	47	58
Supraclavicular		1	4	39	44
Lat fem cutaneous			6	25	31
Interscalene				18	18
PENG				12	12
Classic sciatic			4	6	10
TAP			2	1	3
Ankle			1	2	3
IPAK			2		2
Cervical plexus				1	1
Total	13	132	462	1,238	1,845

**Figure 1:**
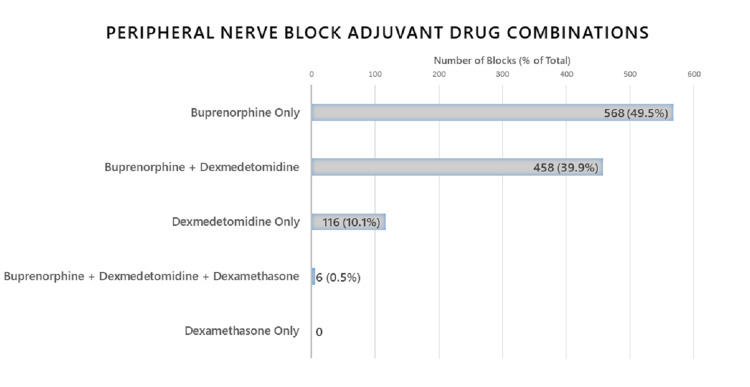
Adjuvant drug combinations used in pediatric peripheral nerve blocks

Buprenorphine, given as a sole adjuvant agent, was the most common regimen, used in 568 blocks (49.5%). The second most common additive regimen was a combination of buprenorphine and dexmedetomidine, which was administered 458 times (39.9%). Dexmedetomidine as a sole agent was used to a lesser extent (116 blocks, 10.1%) and the combination of all three medications (buprenorphine, dexmedetomidine, and dexamethasone) was rarely used (6 blocks, 0.5%). Dexamethasone as a sole peripheral nerve block additive agent was not used in any patient (Figure 1).

Total adjuvant doses are presented in Table 3 as means with one standard deviation. Depending on the surgical case, one to four blocks were performed for each patient’s anesthetic; thus, mean doses for individual blocks are presented as well.

**Table 3:** Doses of adjuvant medications utilized.

Adjuvant	Mean Total Dose Per Patient (Standard Deviation)	Mean Dose Per Block (Standard Deviation)
Buprenorphine (mcg/kg)	3.6 (1.1)	2.8 (1.1)
Dexmedetomidine (mcg/kg)	0.9 (0.4)	0.6 (0.3)
Dexamethasone (mg)	2 (0)	1 (0)

## Discussion

It is common in our institution to place multiple (often up to four) peripheral nerve blocks in one pediatric patient, such as in patients undergoing dual upper and lower extremity orthopedic reconstruction procedures, or extensive bilateral lower extremity surgery. Adjuvant drug dose selection varied, often based on the number of injections. A larger per-kilogram single dose was used if just one block was being performed, and a larger total dose was accepted when multiple blocks were placed.

Despite being the most extensively studied adjuvant [8], clonidine was not used in any peripheral nerve block in the investigation period. It was used by our anesthesiologists initially when adjuvant additions started in 2018, but due to drug shortages at that time, it became unavailable. We switched to dexmedetomidine as additional literature supporting its position as a more effective α_2_-agonist [10] emerged and, as the generic form of dexmedetomidine became available and its cost decreased considerably, our group never returned to clonidine for peripheral nerve blockade. Neuraxial clonidine use during the study period did occur, but was not the route of administration under investigation.

We hypothesize that the presence of a drug reference sheet and medication access played a significant influence on an entire group’s clinical practice. The peripheral nerve block adjuvants reference sheet (Table 1) was printed in bright coloration, laminated, and attached to all ultrasound machines in our department. Buprenorphine was listed as first line as it has provided the most robust prolongation of analgesia duration in the adult literature [13]. The α_2_-agonists were listed as second line, because they have been used most extensively in the pediatric literature [8,9], yet have not been shown to offer as much prolongation of block duration [11]. Both buprenorphine and dexmedetomidine were readily available in the anesthesiologists’ cart, while clonidine and concentrated dexamethasone had to be retrieved from pharmacy. As anesthesiologists became familiar with drawing up the adjuvant medications, it was quickly discovered that the volume ratio of buprenorphine at 3 mcg/kg to dexmedetomidine at 0.5 mcg/kg with our preservative-free, concentrated vials was 1:1. We believe this drug vial availability and ease of use also contributed to the agents’ combined popularity.

A limitation of this retrospective study is its inability to evaluate the efficacy of the adjuvant medication regimens against that of traditional plain local anesthetics. A contributing factor is that our institution often preemptively orders perioperative non-opioid multimodal therapies, such as acetaminophen, non-steroidal anti-inflammatory agents, ketamine, and/or gabapentin. Occasionally even low-dose scheduled opioids were used in anticipation of block regression in the study patients. This aggressive approach to pediatric pain management makes it difficult to interpret a common indicator of efficacy, the time to administration of first analgesic. Studying nurse-reported pain scales is also a nearly impossible proposition, due to the use of multiple pain assessment tools at our institution including the Visual Analog Scale (VAS), the Wong-Baker FACES Scale, the Pain Scale for Intubated Patients (PSIP), the Observational Pain Assessment Scale (OPAS), and the revised Faces-Legs-Activity-Cry-Consolability Scale (rFLACC). To study the efficacy of these adjuvant medications in peripheral nerve blocks, it would require a prospectively designed trial with pre-specified, randomly assigned adjuvant regimens, controlled multimodal pain plans, predesignated pain assessment scales, and be performed in verbal pediatric patients old enough to reliably cooperate with motor and sensory assessments.

An additional constraint of this study is its lack of direct safety data. Specific rates of known adjuvant-related side effects are typically not recorded on the intraoperative anesthetic record and thus could not be investigated here. Only indirect inferences can be made as follows: Since the institution of routine dual/triple antiemetic prophylaxis therapy and an aggressive PONV care set quality improvement project in 2017, we have not had any unplanned admissions for PONV and this did not change with the institution of multimodal peripheral nerve block adjuvants. There were no instances of respiratory depression, as every dose of naloxone administration for this indication is tracked within our institution. No permanent neurologic injuries were reported during this time period as well, though transient neurologic issues related to nerve blocks were not specifically investigated here.

It must be stated that our use of the above multimodal perineural adjuvants is off-label. Moreover, it is important to recognize that the use of bupivacaine in children under the age of 12 years is off-label and that ropivacaine is not FDA approved for pediatric use regardless of age [20]. Yet, these local anesthetics are routinely used in pediatric regional anesthesia practice.

## Conclusions

The intention of this article is neither to delineate efficacy, safety, nor best adjuvant(s). Rather, this report describes one center’s experience with approximately 1,100 pediatric peripheral nerve blocks placed using adjuvant agents. Its purpose is to provide a starting point for medication dosages, and to justify the need for future prospective controlled trials evaluating efficacy and elucidating the ideal combination of adjuvant perineural peripheral nerve block medications for pediatric patients.
